# Safe and effective prescribing with dyslexia

**DOI:** 10.1186/s12909-019-1709-5

**Published:** 2019-07-24

**Authors:** Sebastian C. K. Shaw, Laura R. Hennessy, Michael Okorie, John L. Anderson

**Affiliations:** Department of Medical Education, Brighton and Sussex Medical School, Brighton, East Sussex, UK

**Keywords:** Dyslexia, Specific learning difficulties, Doctors, Prescribing, Medical education, Health education

## Abstract

**Background:**

The term ‘dyslexia’ refers to a condition that impacts upon reading and writing abilities whilst not altering intelligence. Individuals with dyslexia may have difficulties with the speed and accuracy and their reading and writing, amongst other issues.

Dyslexia is not automatically considered a disability but is a protected characteristic under the UK Equality Act (2010), and therefore employers and educational institutions are required to provide ‘reasonable adjustments’ in order to allow individuals to reach their full potential. There is a lack of research on this issue, but what little there is suggests that doctors feel as though any support they received ended when they graduated from medical school.

**Main body:**

A core distinction between medical school and medical practice is the requirement to prescribe medicines as registered medical practitioners. Junior doctors have to master this complex and potentially hazardous skill “on the job”, with a perceived lack of support.

Here, we open up a debate about the potential impact of dyslexia on prescribing, and the need to find supports that may be effective in enabling doctors with dyslexia prescribe medicines safely and effectively – and thus reach their full potential as medical practitioners and promote patient safety.

**Conclusion:**

We argue that medical schools and hospitals could immediately provide dyslexia awareness training in both undergraduate and postgraduate settings. We discuss electronic prescribing systems, and conclude that research is required to identify effective supports for junior doctors with dyslexia.

## Background

Rudolf Berlin, a German Ophthalmologist, first coined the term ‘dyslexia’ in 1887 [1], from the Greek– *dys-* (meaning ‘difficulty’) and *-lexis* (meaning ‘word’) [2]. So, dyslexia means a difficulty with words. However, a contemporary understanding is that dyslexia involves information processing difficulties [3]. It manifests itself as an impairment of reading and/or writing, and does not impact upon intelligence [4]. It is a spectrum of difficulties, which can affect individuals in a wide variety of ways [5]. Some of the difficulties that individuals with dyslexia may experience include [6]:

- Slow reading;
- Slow writing;
- Difficulty with spelling;
- Weak organisational skills;
- Weak short-term memory.

Dyslexia affects approximately 10% of the UK population and is categorised into a group of Specific Learning Difficulties (SpLDs) [3, 7]. In 2007 the BMA reported that 1.7% of entrants to medical schools had an SpLD, and predicted that this number was likely to increase [8]. In fact the number of students disclosing disabilities within higher education (HE) has been increasing [9, 10]. This may be due to efforts to widen access to HE or possibly a reduction in stigma in the HE community as a whole. Generally, it is encouraging to note that support for disabled people in higher education is taken seriously in the UK and globally [11, 12]. However, even though the challenges faced by these students have been highlighted, further research is required to define what supports are effective and how they might be introduced. [9].

Whilst dyslexia is not automatically considered a disability by definition, it is a protected characteristic under the UK Equality Act (2010), and thus employers and educational institutions are required to make ‘reasonable adjustments’ to help individuals to reach their full potential [13]. Within medical schools, reasonable adjustments are usually provided as extra time in exams and extensions to deadlines [14]. However, such supports may vary significantly between different medical schools [15]. Also, individuals with dyslexia report that supports disappear when they progress to the world of junior doctors [15]. There is therefore a question of what adjustments may be pertinent to doctors in the United Kingdom (UK).

*“I think, after medical school, the support is GONE! You are in the real world and people are less sympathetic. You won’t get extra time examining patients… There is no sympathy or empathy and no understanding. And I feel very sorry for dyslexics who aren’t as… who aren’t as high functioning or don’t have as good coping mechanisms as me.”* [15]

Prescribing is the most common healthcare intervention [16]. It is a complex task that every junior doctor will undertake. Safe and effective prescribing is essential for optimal patient outcomes. Prescribing errors can lead to morbidity and possibly mortality. The GMC commissioned EQUIP study highlighted that junior doctors are responsible for most of the prescribing in hospitals and they also have the highest prescribing error rates [17]. However, medical students have not felt adequately prepared for prescribing in the foundation years after graduation [18, 19]. Furthermore, there is seemingly no agreed minimum standard for the teaching and learning of safe and effective prescribing for doctors in training – including junior doctors with dyslexia [20].

In recent years, there has been a renewed emphasis in the teaching and learning of safe and effective prescribing in medical schools and postgraduate training. However, prescribing with dyslexia has not featured prominently. The difficulties associated with dyslexia may negatively impact upon safe and effective prescribing. A study by Wright found that 24% of Deans of nursing and midwifery schools thought that students with dyslexia posed a potential risk of unsafe practice [21]. A solution to this was not offered. Prescribing errors are not unique to healthcare professionals with SpLDs [22], and so it is important to ascertain the true impact of dyslexia.

There may exist important arguments for both supporting junior doctors with dyslexia and preventing patient harm. We approach this debate from a supportive and advocative perspective. Two of the authors of this article have dyslexia (SS and LH). Here, we discuss the potential impacts of dyslexia on safe and effective prescribing and the supports that may be required to help doctors with dyslexia to minimise their risk of prescribing errors – considering both their possible strengths and their potential shortcomings, in the context of a current dearth of research. We therefore also identify areas of research to be addressed.

### Therapeutic decision making

Prescribing and administration of medicines is a multifaceted process. First, the clinical problem(s) for which a prescription is required has to be identified and then an appropriate medicine must be selected. This must then be transcribed into an instruction for the patient to receive the medicine – including a correctly spelt drug name, dose, frequency, and route of administration (e.g. oral). In essence, the finished product must provide all of the information necessary to effectively and safely enable the medicine to be used to treat the patient, without ambiguity. If adequate supports are not in place, dyslexia could significantly compromise this delicate process.

We suggest that dyslexia might influence a doctor’s choice of a medicine. Where multiple medications are available to treat a clinical problem, a doctor with dyslexia who cannot spell a first-line agent might prescribe an alternative with an easier to spell name. There is no published research on this within medicine/medical education, but it poses a potentially significant challenge for the doctor who has dyslexia – and subsequently a potential risk for patient harm. Research is required to identify the challenges and to see how junior doctors with dyslexia cope. Furthermore, supports that could help them cope more effectively need to be determined.

### General handwriting

In clinical practice patient records are still often handwritten [23]. Interestingly, only a small proportion of inpatient prescribing in adult medical and surgical wards in England is performed by electronic prescribing systems [24]. This may prove problematic for doctors with dyslexia.

Dyslexia is known to cause problems with handwriting – not only in the speed of writing, but also its legibility [25]. This can affect the legibility of details of the prescription, including the name of the medicine and the units. On the other hand, doctors with dyslexia may also struggle to interpret others’ handwriting [26]:

*“I am very bad at deciphering other people’s handwriting, I normally ask if there is another FY1 or the nursing staff, I normally have to ask to check things.”* [26]

Handwritten text has been shown to exacerbate dyslexic difficulties [26]. Whilst there is every possibility that there might be others to help in interpretation, the outlook is more uncertain if there is nobody available. SS has experienced occasions where he could not interpret the handwriting of colleagues, and also times where the nursing staff have asked him to interpret others’ entries into the medical notes. What happens when this is a drug chart? For example, ‘does that say 10mg of amlodipine or 10mg of amitriptyline?’ An important distinction.

Therefore, further investigation of electronic prescribing, or even handwriting courses may be needed – there is, at present, no evidence to support or refute either of these. It is also not yet known which of these interventions may be the most beneficial. Based on personal experiences, it is our opinion that electronic prescribing may be a way forward here, as it encompasses so many more factors than just the handwriting of the prescription – for example, possible safety features surrounding allergies and units.

### Spelling of medicines

Many individuals with dyslexia have difficulties with phonological processing and spelling [3, 13]. Some medications can be particularly challenging to spell – “Hydroxocobalamin” (a vitamin B12 injection), for example [27]. In a fast-paced clinical environment which is full of distractions, this could mistakenly become a prescription for “Hydroxychloroquine” (a strong immunosuppressant drug) [28], which could be a catastrophic prescribing error for the patient.

SS has seen a prescription for fluoxetine (an anti-depressant) prescribed as a course of flucloxacillin (an antibiotic) by a doctor – who later corrected their mistake when this was pointed out. Such occurrences could perhaps be minimised through the use of electronic prescribing platforms – whereby the medication names are pre-spelt for the prescribers using the software. It has been postulated that electronic prescribing could potentially reduce the number of drug errors by half [23]. What we do not know is whether there is also a possibility that such systems could promote complacency – and actually cause prescribing errors through the rushed selection of incorrect medications. This is another area in need of further exploration. Could making the process too simple in fact introduce unforeseen issues such as this?

Issues around spelling also highlight the importance of working within an effective multidisciplinary team. For example, having a pharmacist available to ward teams could help to further minimise the risk of this happening. This needs to be tested empirically.

### Documentation of units

Prescribing entails the use of a variety of units. For example: micrograms, milligrams, grams, millilitres, or even ‘units’ themselves. In pharmacology and therapeutics teaching, LH has had teaching on always writing the units of the drugs in full – as is best practice [29, 30]. In doing this, educators aim to promote a safer culture within prescribing– enabling the doctors of tomorrow to reduce the likelihood of misinterpretations. From SS’s experience, however, doctors in clinical practice do not consistently do this. We consider that there may be several possible reasons for this:

- Doctors do not have the time to write them in full;
- The space provided on paper drug charts is insufficient;
- Some may not have been taught to do so;
- It is too difficult for some to do it.

Therefore, a review of current paper drug chart layouts may be advisable, to enable units of medications to be written the full. This is also an instance in which electronic prescribing systems might again prove beneficial – reducing the risk of errors of interpretation around units.

### Personal coping strategies

It is well documented that individuals with dyslexia develop coping strategies to overcome their dyslexic difficulties [31–33]. These may include double-checking or the use of electronic devices [26]. Coping strategies could be investigated to share positive experiences and lessons. These might also be encouraged within relationships with mentors [34].

Much of the current research into dyslexia within healthcare has been undertaken within nursing [10, 21, 22, 34–38]. The Royal College of Nursing has published a toolkit on dyslexia and other SpLDs [39]. This explains how employers and employees can help themselves to overcome difficulties. For example, getting a colleague to check your calculations and learning more about the drugs that are frequently used within the area in which you work [39].

Due to increasing work demands there is currently a high vacancy level [40]. Therefore, departments have to function with a suboptimal number of trained staff and there may not be the capacity for a doctor with dyslexia to ask a colleague to double-check their work. However, nursing students and other professionals report that the most appropriate action to reassure themselves (and others) is to check, double-check and, in some situations, triple-check their own work [39]. The counter-argument here is that this takes extra time in situations with already-overburdened staff. This seems an approach worth investigating to overcome the issue of involving others.

### Potential supportive adaptations to the workplace and training

*“Reasonable Adjustments should be put in place as soon as possible. Failure to implement Reasonable Adjustments would be a breach of the Equality Act.”* [41].

Previous research has shown that dyslexia may foster negative emotional responses in junior doctors with the condition [15]. This has also been demonstrated in the case of a medical student with dyslexia [31]:

*“I felt that those around me were fed up with me, thought I was inept or, even, hated me.”* [31].

Such stress exacerbates individuals’ dyslexic difficulties [41] which may be further exacerbated by a perceived lack of personal support [42]. By this logic, personal or “pastoral” support could be vital to help overcome prescribing difficulties.

There is also a need to educate non-dyslexic doctors about dyslexia, in order to dispel any myths and stigmas surrounding the condition – to further improve the working life of these doctors.

*“Most people (doctors) don’t see it as a real… They don’t see it as a real disease. They just see it as made up”* [15].

This is supported by the British Dyslexia Association, who state that “dyslexia awareness training is essential” in order to provide adequate support – through the education of colleagues and managers [41]. From our experience, dyslexia education is largely lacking within undergraduate and postgraduate medical education. This is an area that therefore needs to be investigated further. We suggest that this could be achieved through teaching on dyslexia and other SpLDs at medical school and during early Foundation training. In doing so, it is possible to demystify dyslexia, thus fostering a more positive and open culture within our health service. This is supported by prior research, where participants felt that a negative attitude towards dyslexia in medical education was fostered by a lack of understanding [15]. Again, this stands against arguments that the medical curriculum is already over-full, and that in-service training puts increased pressure on staff.

External organisations could provide dyslexia awareness training [43], and thereby healthcare organisations could achieve greater normalisation of the condition. This could also ease disclosure amongst those with dyslexia – thus enabling individuals to better access any support offered.

### Prescribing tips for doctors with dyslexia

Through the synthesis of our experiences in addition to the current literature, we offer the following tips to doctors (or medical students) with dyslexia who might be nervous about prescribing:

- Try to prescribe right away – unless you feel you need to go away and do it later!
- Do not rush in the writing of the prescription itself. You may feel pressured by those around you on jobs. Take your time, to ensure that you prescribe safely, regardless of the reactions of others.
- Always double-check or even triple-check your work before leaving.
- Look up any medicines that you are not completely sure of – utilise appropriate sources of information.
- Always write units in full on handwritten drug charts.
- If in doubt, ask someone.

## Conclusions

Prescribing is a complex skill that is largely mastered in the training years. Supports should be considered for the early training years of clinical practice for junior doctors with dyslexia. We have discussed various strategies to help doctors with dyslexia, such as:

- Dyslexia awareness training in order to foster a positive and open culture – thereby reducing stress on doctors with dyslexia. We argue that this support and awareness may help to reduce the risk of patient harm.
- A possible increase in electronic prescribing.
- Additional supports for doctors with dyslexia – which may need to be tailor-made and specific to individuals.
- We propose that further research is needed to accurately identify the best possible solutions – to identify:
- What “supports”/coping strategies are used by junior doctors with dyslexia;
- How effective these are;
- What (other) supports might be needed.

We are planning a series of studies to address some of these issues – to identify problem areas; to investigate what supports/coping strategies are currently employed; and to evaluate the effectiveness of different supportive/coping measures. As a first stage, we (JA, SS & MO) have conducted a survey, exploring these aforementioned areas (currently in preparation). We welcome expressions of interest from other researchers on future collaborations in this area.

Ultimately, the workplace should be optimised for a doctor with dyslexia and this programme might lead to improved patient outcomes.

## Abbreviations

NHS: National Health Service
SpLDs: Specific Learning Difficulties
UK: United Kingdom
